# Transactions of the Chicago Society of Physicians and Surgeons

**Published:** 1875-04-01

**Authors:** Ralph E. Starkweather


### Society Reports.

### TRANSACTIONS OF THE CHICAGO SOCIETY OF PHYSICIANS AND SURGEONS.

### Regular Meeting, March 22d, 1875.

*Reported by Ralph E. Starkweather, M.D.*

THE Society met as usual at the Grand Pacific Hotel, the President, Dr. Bartlett, in the chair.

Dr. Etheridge read the following report from the Committee on Therapeutics:

#### ERGOTINE.

Dr. E. R. Squibb, of Brooklyn, submitted some notes to the American Pharmaceutical Association in 1873, which are published in the Association's proceedings for that year, on Ergot and Ergotine. After speaking of the market value of ergot crude, and its pharmaceutical manipulations, he speaks as follows of ergotine, so called:

"The existence of one or more active principles, which may be isolated for therapeutic use, has been very generally believed, and various substances have been extracted and sold under the name of ergotine—those of different makers having different properties and different doses, but all producing some of the effects of ergot, though in different degrees. After a careful attention to the literature of this subject, and comparing it with the results which seem to have been realized in practice by experience, the writer does not believe that there is an active principle separable from ergot, which, in any proper sense, represents the drug. Like senna, rhubarb, and many other drugs, its effects seem to be dependent upon a natural association of its various separable elements. If this be true, there is no such thing as ergotine, and the various substances so called only represent the activity of the drug, as they represent more or less perfectly its entire composition. Of course, certain of its constituents, such as lignin, starch, fixed oil, gum, etc., are known to be inert, and such being excluded by choice of a proper menstruum for extracting it, the nearer its preparations come to representing or containing all the remainder of the drug the better."

#### ERGOT—AQUEOUS AND ALCOHOLIC SOLUTIONS OF.

These two differ in their effects on the circulatory apparatus. The aqueous solution excites the activity of the cardiac inhibitory centres, and the vaso-motor centre in the medulla; it slackens the pulse, narrows the calibre of the small arteries, and increases the blood-pressure. Very large doses paralyze the heart at once, and so effectually that the induced current fails to restore it. These effects on the heart and vessels are not at all produced by the alcoholic solution.

Thel atter produces symptoms akin to those of æro-narcotic poisoning, viz.: irritation of the mucous membrane of the stomach and bowels, tonic cramps, and agitative violent spasms—symptoms which cannot be produced by aqueous solutions of ergot.

ERGOTINE, IN VARICOSE VEINS.

Dr. Paul Vogt, assistant surgeon in the out-patient clinic at Greifswald, first tried ergotine injections for varicose veins. In the *Berliner Klinische Wochenschrift* for March 4, 1872, he first reports the use of this drug as above indicated. An extensive varix, which had occupied the leg for years, yielded in the course of a week to two injections. Subsequently there followed diffuse light inflammation, leaving a hard, circumscribed infiltration, which eventually subsided.

In many other cases he has used, in a similar manner and with as remarkable result, as in varicocele, hæmorrhoids, certain forms of angioma, telangiectasis, etc.

The most generally-accepted idea is, as to the modus operandi of ergot subcutaneously, that diminution of the calibre of vessels is produced by the drug stimulating the smooth muscle of the veins and arteries to contract. In its use in varicose veins, it doubtless partially produces diminution of the capacity of the veins, by producing the inflammatory infiltration above mentioned, which directly compresses the veins.

In the same periodical above mentioned for August, 1874, Dr. Max Schuler stated, as a result of many experiments, that ergot produces contraction of arterioles. The smaller the vessel, the longer and firmer is the contraction. Arteries increase proportionally in smooth muscular fibre as they become smaller. From this fact it is inferred that ergot exerts its effect on this muscular fibre, *not* necessarily through the vaso-motor nerves, for the same effect—viz., vascular contraction—could be secured in a part injected with solution of ergot after the sympathetic nerve supplying the part was cut.

So firm indeed is this contraction, that it cannot be changed by persistent inhalations of nitrite of amyl.

From a knowledge of this effect of ergot on the smaller vessels, to the use of this medicament in hæmorrhages, as epistaxis, hæmatemesis, hæmoptysis, hæmorrhage from the bowels, kidneys, etc., is but a step.

A close observer of medical literature sees, of late months, very many accounts of the use of this drug in these troubles hypodermically. Ergot by the stomach will produce none of the effects that can be called hæmostatic; subcutaneously, it will.

Dr. Drasche, of Vienna, reports many cases of hæmatemesis being treated with subcutaneous injections of ergot. The point of selection was the neighborhood of the pectoral muscle. Sometimes more than one injection was necessary. After the first one there is always a diminution, and, in many cases, a cessation of bleeding.

One phthisical young man, who had had violent epistaxis every morning for a week, and had been unsuccessfully treated with ice and perchloride of iron, was completely cured after two one-grain injections of ergot.

In most cases of hæmatemesis ergot was used after the ordinary means had been used in vain, and the checking of the hæmorrhage was prompt.

It was successful after ergot had been used in large quantities in the stomach.

In hæmorrhages in scorbutus ergot has been successfully used, though the amount and duration of the local irritation were considerable.

SULPHIDE OF CARBON IN THE TREATMENT OF ATONIC ULCERS (SORES) AND CHRONIC ULCERATIONS.

[*By Paul Guillaumet, Interne of St. Lazare.*]

"If there is any affection upon which the therapeutical armamentarium has been largely used from time immemorial with very variable success, it is certainly chronic ulcers.

As I have thought that there is a certain interest attaching to any new remedy addressed to these obstinate affections, I desire to speak of the local application of the sulphide of carbon in ulcerations, differing in characteristics and origins, but presenting, all of them, common features, viz., *atony* and *chronicity*, with a tendency to extension, and a total absence of reparative nature and cicatrization."

This agent was used thus in ulcerations first, in 1867, by M. Michel, when he was an interne in Saint Lazare. Its abominably nauseous odor, and its toxic action are the predominant characteristics of carbon sulphide, which have doubtless kept it from extended use.

The German physicians have used this agent in arthralgias and neuralgias successfully, probably through its refrigerant and anæsthetic action. Compresses soaked in carbic sulphide in some aromatic essence were placed over the painful part, and renewed from time to time till relief followed. Krimmer used it in the treatment of white swellings. Ewenbull used its vapors mixed with those of iodine in certain affections of the eye and ear. It has also been used upon the erysipelatous redness of chilblains. These constitute the only uses, externally, to which this agent has been devoted.

The writer details three cases, selected from a large number thus treated, one of which is here given in full.

"CASE I.—*Large ulceration of the right labium majoram, of four and a half months' duration, in a syphilitic subject. Cure in fifteen days.*

Alice V., æt. 23, admitted May 25, 1874. Upon the inner surface of the right labium majoram, extending a little to the outer surface, she has a large ulceration, extending nearly the whole length of the labium, measuring three centimetres in breadth. It is atonic, has a grayish base, with irregular serrated borders, and has a depth of about four to five millimetres.

The patient evidently has syphilitic antecedents. As to the subject of the origin of this ulcer, she says that she experienced a severe itching produced by some small pimples, grouped together in that region, and that she scratched them with her finger-nails and produced an excoriation, which, irritated by frictions of the tight clothing, became inflamed and produced an ulceration. For some days subsequently she paid no attention to it, but seeing it increase daily instead of diminishing, she concluded to use some poultices and cerate. Then she visited St. Louis Hospital, and was regularly cauterized every eight days with various caustics.

When she entered this hospital she had had this ulceration four and a half months, without its being in the least diminished. From this time the sulphide of carbon was used and with success as complete as unexpected. Thirteen applications in *fifteen days* sufficed to produce complete cicatrization of the ulcer."

Sulphide of carbon has been used with good results in the treatment of syphilitic ulcerations, ulcerating tumors, ulcerations of the neck, of the vagina, of the vulva, corroding ulcers, ulcerations in scrofulous subjects, etc.

After the first applications of this agent there is no noticeable change in the aspect of the ulcer. After the fifth or sixth application a sensible amelioration and a very rapid course to cicatrization follow. The progressiveness of improvement is always so characterized by regularity that, by comparing a portion of the raw ulcer with a portion already healed over, the physician is enabled to predict exactly the number of applications still necessary to complete a cure.

It must be used with care because of its extreme volatility and nauseous odor. The method of applying it is as follows, according to the author: The bottle containing *pure* sulphide of carbon is held very near to the ulcer to be dressed; a piece of charpie is soaked in the liquid, and squeezed upon the mouth of the bottle to expel any excess of the drug; then the piece of charpie is lightly and rapidly brushed over the surface of the ulcer, which is immediately covered with a powder of subnitrate of bismuth, or of starch. These powders, inert of themselves, play a double part: they isolate the labia (when one of them is the seat of the sore), and diminish evaporation of the medicine which is accelerated by the body heat.

The writer thinks that its action is very obscure. It coagulates the albumen of the ulcers, and it seems neither to hasten nor retard fermention. He says the following changes occur after applying the medicament: "A change of color of the tissues occurs; this modification, which is not very evident in pale ulcers, is plainly shown on those whose granulations are active; the tissues become pale. This decoloration is not of long duration. At the end of a certain time it disappears, to be replaced by a very red blush. This is very easily demonstrated upon granulating ulcers. Then comes the pain; a pain almost instantaneous, very severe, but very fugacious. I have seen in certain subjects at this time a period of anæsthesia succeed which lasted several hours, whilst the painful period does not exceed twenty to sixty seconds in most patients. This pain, very acute after the first few applications, diminishes in a few days, to be almost none at all after the last applications. As a rule, it may be said that its intensity is in inverse ratio to the number of the dressings."

"The action of this medicament is so rapid upon recent sores that after the first or second dressing a change is readily noticeable. It is much less rapid upon very old ulcers, at least after the first applications. It is not rare to dress an old sore five or six times before obtaining an appreciable modification. But from the day that a change is noticed the process of cicatrization is very rapid. The amount of the sulphide to be used having a great influence upon the results to be obtained, should be proportional to the ancientness and vitality of the ulcer. In using a rather large dose of carbon sulphide, I have not had any scarring follow, nor poisoning accident if minims were used, but this has seemed to retard rather than accelerate cicatrization."

In conclusion, the author summarizes the principal characteristics of this agent in the following propositions:

1. "Sulphide of carbon is a powerful cicatrizer.

2. "Its action is limited and rapid. It is wholly local, and does not produce a single accident which follows the inhalation of its vapors.

3. "Its application is accompanied with a pain sometimes very severe, proportional to the susceptibility of the patient, but of a very short duration in most patients, followed immediately by a period of anæsthesia which is not constant.

4. "Sulphide of carbon acts upon ulcers of various kinds, (syphilitic, scrofulous, diphtheritic, etc.,) and changes them all beneficially.

5. "This is a valuable agent in the treatment of wounds and ulcers, presenting the common characteristics of *atony* and *chronicity*."

Dr. Bevan, from the Committee on Clinical Reports, next read the following notes of cases occuring under his care in Cook County Hospital:

CHRONIC MYELITIS.

Peter Lorentzen, age 50; laborer; Dane; admitted December 4th, 1874. The patient states that he was well until a year ago last October. While working on a ladder, he slipped, and fell some sixteen feet, striking on his back. He was confined to his bed four months, when he was again able to resume business. After working about three months, he experienced a recurrence of the pain in the side and back. This soon became so severe, that he again discontinued work. He came to the hospital last June, remaining under treatment some five weeks, and again returned home. Ever since this time, the pain has steadily increased. For the past four weeks, he has been much worse; he has been unable to walk; says his legs are getting weak, and complains of sharp, stitching pain, shooting toward the extremities. Appetite has always been good. Bowels inclined to constipation.

*On admission*, patient somewhat emaciated; face, anxious; has the appearance of suffering; feels more comfortable when the thighs are flexed.

*On examination*, no tenderness along the spine; no swelling or pain in any of the joints, although the patient thinks he is suffering from rheumatism. In walking, he has to be supported, and holds the lower portion of the spine in a fixed position, and has a kind of rotary gait. The left hip is prominent, the trochanter apparently standing out. Pressure, however, does not cause pain. He is ordered pot. iodid. gr. vij, and strych. sulph. gr. $\frac{1}{30}$, in a simple bitter tonic, three times a day.

Dec. 18th.—Patient sleeps poorly at night; the pain seems to be worse.

Jan. 2.—The patient still complains of severe pain in left lumbar region. Sleeps poorly at night. Is becoming more emaciated. Appetite pretty good; bowels costive.

Feb. 1.—Patient thinks he has not improved any since he came into the house. Cannot stand or walk. Has constant and severe pain in the left leg, from the hip to the knee, interfering with sleep.

Feb. 4.—The potass. iodid. and strychnia were discontinued, and instead, fl. ext. ergota ʒ ss., three times a day, and a mixture of equal parts tr. iodini and glycerine, to be applied over the spine and sacrum.

Feb. 15.—Patient has continued the use of the last prescription up to this time. He says he has a good deal less pain; sleeps well; has a good appetite; and, in fact, feels much improved. Pretty fair motion of the legs.

March 1. — Continued improvement.

SUBACUTE MENINGITIS.

Alice Morris, aged 21; laundress; native of United States; admitted January 19, 1875; died Feb. 4, 1875.

About three months ago, patient was working in a laundry as ironer, in a damp basement where there was no fire. In changing her irons, she was compelled to go from this cold room into the drying room, which was always kept at a high temperature, thus constantly exposing herself to extremes of temperature. She had been working thus about four months, when she began to have pain in the head, nausea, and vomiting. Would wake up in the night with an attack of pain in the head; would eject the contents of the stomach and feel relieved. The attacks were most apt to occur about 3 o'clock, A.M.

The pain in the head constantly increased; became throbbing in character. She soon had to discontinue all work, and would sit around for several hours, stupid with pain. The sight became impaired; light was painful; luminous objects seemed to float before the eyes; smell and taste were both partially lost. All food had an acid taste. Bowels costive, moving once or twice a week. She has a severe shooting pain in the back of the neck, aggravated by motion or pressure. Sleeps poorly. Is dizzy, and unable to walk unsupported. She can scarcely remember anything, even common events.

*On admission*, patient poorly nourished; skin normal; tongue flabby and tremulous, covered with brownish fur. Prefers to lie on the side, with the limbs drawn up, and eyes shaded from the light. Slight movements of the head or neck cause facial contortions, indicative of severe pain.

Pulse 50. Resp. 22.

| | |
|---|---|
| ℞.—Potass. brom., | gr. xv. |
| Strychnia sulph., | gr. 1–30. |

Three times a day.

The progress of the case was steadily downward.

Feb. 1.—Patient seems to be in an almost comatose condition. She is roused with difficulty, and cannot make an intelligent response to questions. Urine and fæces pass unconciously. Takes no nourishment; will not swallow, but allows liquids to run out of the mouth. Pupils dilated.

Feb. 4.—*Died.*

The autopsy revealed some tuberculous deposit in the lungs. On opening the brain-box, several ounces of clear serum escaped from beneath the membranes of the brain and cord. Other organs healthy.

EPILEPSY.

CASE 1.—Wm. Hutchinson, aged 24; bar-tender; native of United States; admitted January 27, 1875; discharged February 6, 1875.

Patient was always well until he was nine years of age, when he was struck upon the head with a hatchet. Does not remember whether he was much affected by it at the time or not. (States that at the age of twelve, he was bitten by a mad dog). His health was good until eight years ago, when, while walking along the street on a very hot day, he suddenly fell, and was unconcious for an hour or two. During the next two days he suffered from several such attacks. During this time he had very severe pain in the head. One month later, he had a recurrence of the same kind of attacks, and the pain in the head was present also.

He enjoyed immunity from attacks until one and a half years ago, when he went through precisely the same experience. At this time, he was sick for two weeks, suffering from pain in the head, and occasional loss of conciousness. His friends said he was convulsed during the attacks, requiring several men to hold him. He has been a steady drinker for several years until last Christmas; since then he totally abstained until a week ago Monday, when he began to drink very hard for two days only. Last Saturday, he began having pain in the head and feeling drowsy. This continued increasing until the convulsive attacks returned again. He was brought here suffering from those attacks. Ordered

| | |
|---|---|
| Pot. brom., | gr. xv. |
| Chloral hyd., | gr. xv. |

Every three or four hours.

Feb. 2.—Patient seems to be improving slowly. The attacks are less frequent. He is ordered

| | |
|---|---|
| Pot. brom., | gr. xx. |
| Fl. ext. valerian, | ʒ i. |

Three times daily.

Feb. 6.—Thinks he feels well enough to go home, and is discharged.

March 4.—One month later, patient was brought in with another attack similar in character. He is put upon pot. brom. gr. cxx. in the twenty-four hours, with the effect of speedily reducing the number of seizures and the cephalalgia.

Case 2.—Emma Rauworth, aged 35; housewife; native of England; admitted February 6, 1875.

For the past seventeen years, patient has had occasional epileptic attacks, not traceable to any exciting cause, as the patient occasionally goes six or eight months without a fit, and then may have two or three at short intervals. Has had five children—three alive now, and healthy; two died in infancy. She menstruates regularly and easily.

Six weeks ago, the patient began to show signs of the present attack. She would sit down near the fire and drop into a partially insensible condition, resembling sleep, from which it would be impossible to rouse her. She would enjoy her natural slumber at night; would rouse up about 5 A.M.; soon after, would go into this stupor, and remain so until 3 or 4 o'clock P.M. On awaking, she seems trying to escape some imaginary danger, but in a few moments seems to revive and converse naturally. During the sleep, she does not know anything about what transpires. She has been moved from place to place, even several miles in a buggy, without the slightest knowledge of the event. She has lately complained of pain and sense of weight in the top of the head. She has lost flesh; never asks for food, but will take it, if offered, when awake. Bowels move regularly each day, and urine passes normally. She attends to these calls herself.

10 A.M.—She is now lying quietly in stupor; has been for four or five hours; breathes easily. Pulse 74; resp. 12. Cheeks a little colored; muscles flaccid, with occasional twitching; jaws firmly closed; eyelids resist efforts to open; eyes rolled upward.

Feb. 10.—℞.—Pot. brom., gr. xx., three times a day.

11.—Had a well marked epileptic fit yesterday, but of short duration.

Increased the dose to gr. xxx., three times a day.

12.—Feels better; sleeps pretty well now most all night.

15.—Instead of a stupor, the patient now experiences a pain in the head, about 5 A.M.

20.—The pain is growing less.

21.—Was religiously excited, and is a little worse.

30.—Better again.

March 2.—Is ordered as a tonic,

| | |
|---|---|
| Strych. sulph., | gr. 1–60. |
| Ac. nit. dil., | gtt. v. |
| In the infus. gent., | ℥ ss. |

After meals.

During the discussion of the above papers, Dr. Bevan mentioned a case of diabetes mellitus which he had treated with good results by the use of the fluid extract of ergot, in doses (finally) of one fluid drachm four times a day. The amount of urine first voided was sixteen pints daily. Ten weeks after beginning treatment the amount of urine daily voided was only four pints, and very much less saccharine; the thirst and emaciation of the patient had become much less.

Dr. Owens queried whether there was any disease ergot would not relieve, and quoted a case of diarrhœa of several months' duration (at least seven months) which he had relieved by the use of wine of ergot. In regard to the sulphide of carbon, his experience in its use upon ulcers had been satisfactory. One of his patients had had an ulcer upon each leg. He treated one of them by elevating the limb and warm water dressings; the other he treated with the sulphide of carbon, and healed the ulcer quickly. The time lost by the first method of treatment was over two weeks.

The Secretary, Dr. Hyde, thought that in many instances chronic ulcers could be treated in no way more efficacious or expeditious than by cross-strapping and the starch bandage.

Dr. Powell mentioned two methods of treating ulcers of the leg: one by transplantation of the skin; the other by half-inch incisions, at intervals, around the entire ulcer. It would not be sufficient merely to remove and use epithelial scales—actual integument was needed. A piece the sixteenth of a square inch would suffice to make a dozen insertions, each of which would make a centre for granulations which would cover about an inch. Upon the coalescence of these islands of healthy skin, the ulcer would be healed. Little cuts should be made in the floor of the ulcer, and a piece of sound skin inserted.

Dr. Hollister adverted to a method formerly used by Dr. Brainard, that of placing upon the ulcer a piece of lint sprinkled with a little iodine.

Dr. Hyde had had good results in cases of ulcers from indolent buboes, in the employment, after the above method, of bromine.

Dr. P. S. Hayes said that in one of the preparations of ergot, acetic acid was used, about one per cent. being added to the officinal fluid extract, which rendered the liquid preparation more permanent; the acid has the effect of removing all inert matter. Ergotine is not a simple body like the morphia sulphate.

Dr. Powell, in a case of double popliteal aneurism, had employed hypodermic injections of ergotine upwards of five weeks with no good results—one of the tumors even enlarged.

Dr. Etheridge remarked that in treating the varicose veins the injections of ergotine would be useless unless made near the varix. The local inflammation produced by the irritating alcohol might be sufficient to diminish the tumor. He never recommended the alcoholic solution for hypodermic injections; the alcoholic and aqueous solutions have no one effect in common. One grain of the aqueous extract represents one minim; this, properly diluted, may be used for hypodermic injection.

The discussion was continued to a late hour by Drs. Davis, Merriman, Henrotin, and others, accompanied by reports of cases.

